# Comparison of various techniques for cataract surgery, their efficacy, safety, and cost

**DOI:** 10.4103/0974-620X.71880

**Published:** 2010

**Authors:** Parikshit Gogate

**Affiliations:** Gogate’s Eye Clinic, Pune and Lions NAB Eye Hospital, Miraj, India

Cataract is the commonest cause of avoidable blindness worldwide,[1] and cataract surgery is the commonest procedure performed in ophthalmology. Cataract surgery is also one of the most cost-effective surgical interventions in terms of the quality of life restored.[2] It was the most common surgical procedure performed in the United States in the past decade,[3] and formed the major source of earning for many ophthalmologists. It is fast, relatively risk free, does not need admission or general anesthesia and yet gives dramatic recovery compared to the preoperative condition. This makes it the favorite of not just operating surgeons, but also of policy makers, health managers, and hospital administrators.

Techniques of cataract surgery have changed dramatically in the past three decades. Sir Stewart Duke Elder mentioned intracapsular cataract extraction (ICCE) as the surgery of choice in his venerable tome in 1967 and was not impressed by the new technique called phacoemulsification (Phaco).[4] That would be hearsay today.

The Madurai intraocular lens implant study demonstrated for the first time the superiority of extracapsular cataract surgery (ECCE) with posterior chamber intraocular lens implantation (PCIOL) over ICCE.[5] Both resulted in a similar best-corrected visual acuity, but ECCE–PCIOL had fewer complications and resulted in a better uncorrected vision. The difference was more marked when quality of life and vision function were considered.[6] At about the same time, a randomized control trial comparing Phaco with ECCE found Phaco to be more effective and economical in the United Kingdom (UK).[7] Phaco soon replaced ECCE and formed the major proportion of the cataract surgeries in the UK.[8]

Manual small incision cataract surgery (SICS) in which the nucleus was prolapsed through a self-sealing scleral tunnel was developed in the United States and Israel and later popularized in India. As it was found to be safer, more effective and cheaper than ECCE, it became popular in India and forms the major proportion of the cataract surgeries done in south Asia.[9,10] Manual SICS was considered a poor cousin of Phaco till a randomized control trial demonstrated it to be not only economical,[11] but almost as effective as phacoemulsification.[12,13] There is a difference in 0.3–0.5 diopter of astigmatism between SICS and Phaco, but a substantial difference in cost. Should a large cataract surgery program bear this cost for the small incremental benefit?[14] A version of SICS is being taught and popularized the world over by major international non-governmental developmental organizations. While it may be possible to have small incision 99.9% of the times,[15] some cataracts such as grossly subluxated lenses, very hard cataracts, or those with poor endothelial counts are better removed through a larger incision.

Phacoemulsification with foldable IOLs is undoubtedly the gold standard wherever Phaco machines and trained surgeons are available and the service affordable. Unfortunately, the technique depends upon not only just a costly piece of technology, but also on more expensive consumables and trained human resource. Cataract surgery has become a refractive surgery today as patients demand better and earlier visual rehabilitation. A patient undergoing ICCE would require +10.0 + 1.5 × 180° spectacle correction for distance. ECCE + IOL implantation has done away with the spherical correction and Phaco with the cylindrical correction, but the patient still needs an optical aid for near vision.

Multifocal IOLs are pushing the envelope by giving unaided, not just distance, but also clear near vision.[16] Newer materials, lens designs, and placement techniques contribute to making the pseudophakic eye function almost like a young clear lens phakic eye.

A study comparing bimanual Phaco (Phaconit) with co-axial Phaco had found a small, but significant difference in the astigmatism.[17] At present, it does not seem to offer a significant advantage over the existing technique unless sub 1 mm incision IOLs become available.

Despite numerous advances, results of cataract surgery still vary the world over.[8,18,19] While surgeons remain obsessed by techniques, patients are more concerned about the outcome. Even countries that have the best possible infrastructure and human resource still have some patients waiting for cataract surgery.[20] A diligent preoperative evaluation, proper asepsis, precise intraoperative skill, and good postoperative care are essential to have repeated good outcomes. While most factors remain the same, what makes the most difference in outcome is the skill, patience, and competence of the surgeon. While we laud the many technical advances that have happened in this realm, we should not forget that the surgeon who holds the instrument is still the most crucial factor in the success of cataract surgery.

## References

[1] Resnikoff S, Pascolini D, Etya’ale D, Kocur I, Pararajasegaram R, Pokharel GP (2004). Global data on visual impairment in the year 2002. Bull World Health Organ.

[2] Porter R (1998). Global initiative: The economic case. Community Eye Health.

[3] Ellwein LB, Urato CJ (2002). Use of eye care and associated charges among Medicare population 1991-1998. Arch Ophthalmol.

[4] Duke-Elder S (1969). System of Ophthalmology. Diseases of lens and vitreous; glaucoma and hypotony.

[5] Prajna NV, Chandrakanth KS, Kim R, Narendran V, Selvakumar S, Rohini G (1998). The Madurai Intraocular Lens Study.2: Clinical outcomes. Am J Ophthalm.

[6] Fletcher A, Vijaykumar V, Selvaraj S, Thulasiraj RD, Ellwein LB (1998). The Madurai Intraocular Lens Study.3: Visual functioning and quality of life outcomes. Am J Ophthalmol.

[7] Minassian DC, Rosen P, Dart JK, Reidy A, Desai P, Sidhu M (2001). Extracapsular cataract extraction compared with small incision surgery by phacoemulcification: A randomised trial. Br J Ophthalmology.

[8] Jaycock P, Johnston RL, Taylor H, Adams M, Tole DM, Galloway P (2009). UK EPR user group. The Cataract National Dataset electronic multi-centre audit of 55,567 operations: Updating benchmark standards of care in the United Kingdom and internationally. Eye.

[9] Gogate P, Deshpande M, Wormald R, Deshpande R, Kulkarni S (2003). Extra capsular cataract surgery compared with manual small incision cataract surgery in community eye care setting in western India: A randomized control trial. Br J Ophthalmol.

[10] Gogate P, Deshpande M, Wormald R (2003). Is manual small incision cataract surgery affordable to developing countries? A cost comparison with extra capsular cataract extraction. Br J Ophthalmol.

[11] Gogate P, Kulkarni S, Krishnaiah S, Deshpande R, Joshi S, Palimkar A (2005). Safety and efficacy of phacoemulsification compared with manual small incision cataract surgery by a randomized control trial: Six weeks results. Ophthalmology.

[12] Gogate P, Deshpande M, Nirmalan P (2007). Why do phacoemulsification? Manual small incision cataract surgery is almost as effective and more economical. Ophthalmology.

[13] Ruit S, Tabin G, Chang D, Bajracharya L, Kline DC, Richheimer R (2007). A prospective randomized clinical trial of phacoemulsification vs. manual sutureless small-incision extra capsular cataract surgery in Nepal. Am J Ophthalmol.

[14] Thomas R (2009). Role of small incision cataract surgery in the Indian scenario. Ind J Ophthalmol.

[15] Thomas R, Kuriakose T, George R (2000). Towards achieving small-incision cataract surgery 99.8% of the time. Ind J Ophthalmol.

[16] Avitabile T, Marano F (2001). Multifocal intra-ocular lenses. Curr Opin Ophthalmol.

[17] Cavallini GM, Campi L, Masini C, Pelloni S, Pupino A (2007). Bimanual microphacoemulsification versus coaxial miniphacoemulsification: A prospective study. J Cataract Refract Surg.

[18] Huang W, Huang G, Wang D, Yin Q, Foster PJ, He M (2010). Outcomes of cataract surgery in urban southern China: The Liwan Eye Study. Invest Ophthalm Vis Sci.

[19] Abdelmoaty S, Behbehani AM, Aljazzaf A, Grigis N, Eslah E, Marouf T et al (2006). The Kuwait cataract outcome study: A 12-month evaluation. Med Princ Prac.

[20] Khandekar R, Mohammed AJ (2004). Cataract prevalence, cataract surgical coverage and its contribution to the reduction of visual disability in Oman. Ophthalm Epidemiol.

